# Clinically relevant dual probe difference specimen imaging (DDSI) protocol for freshly resected breast cancer specimen staining

**DOI:** 10.1186/s12885-021-08179-8

**Published:** 2021-04-21

**Authors:** Broderick J. House, Marcus J. Kwon, Jasmin M. Schaefer, Connor W. Barth, Allison Solanki, Scott C. Davis, Summer L. Gibbs

**Affiliations:** 1 Biomedical Engineering Department, Oregon Health & Science University, 2730 S Moody Ave, Mail Code: CL3SG, Portland, OR 97201 USA; 2 Thayer School of Engineering, Dartmouth College, Hanover, NH 03755 USA; 3 Knight Cancer Institute, Oregon Health & Science University, 2730 S Moody Ave, Mail Code: CL3SG, Portland, OR 97201 USA

**Keywords:** Dual probe difference specimen imaging, Breast conserving surgery, Tumor margin assessment, Fluorescence guided surgery, Paired agent imaging

## Abstract

**Background:**

Re-excision rates following breast conserving surgery (BCS) remain as high as ~ 35%, with positive margins detected during follow-up histopathology. Additional breast cancer resection surgery is not only taxing on the patient and health care system, but also delays adjuvant therapies, increasing morbidity and reducing the likelihood of a positive outcome. The ability to precisely resect and visualize tumor margins in real time within the surgical theater would greatly benefit patients, surgeons and the health care system. Current tumor margin assessment technologies utilized during BCS involve relatively lengthy and labor-intensive protocols, which impede the surgical work flow.

**Methods:**

In previous work, we have developed and validated a fluorescence imaging method termed dual probe difference specimen imaging (DDSI) to accurately detect benign and malignant tissue with direct correlation to the targeted biomarker expression levels intraoperatively. The DDSI method is currently on par with touch prep cytology in execution time (~ 15-min). In this study, the main goal was to shorten the DDSI protocol by decreasing tissue blocking and washing times to optimize the DDSI protocol to < 10-min whilst maintaining robust benign and malignant tissue differentiation.

**Results:**

We evaluated the utility of the shortened DDSI staining methodology using xenografts grown from cell lines with varied epidermal growth factor receptor (EGFR) expression levels, comparing accuracy through receiver operator characteristic (ROC) curve analyses across varied tissue blocking and washing times. An optimized 8-min DDSI methodology was developed for future clinical translation.

**Conclusions:**

Successful completion of this work resulted in substantial shortening of the DDSI methodology for use in the operating room, that provided robust, highly receptor specific, sensitive diagnostic capabilities between benign and malignant tissues.

## Background

With more than 284,000 expected new breast cancer diagnoses in 2021 alone, breast cancer is the second leading cause of cancer related deaths for women in the United States (US) making early, and precise, treatment imperative [1]. Currently, the standard of care for the treatment of early stage breast cancer is breast conserving surgery (BCS) followed by adjuvant therapy [2, 3]. The success of BCS, however, is reliant upon negative or clear margins, with positive margin status thought to be a primary indicator of local breast cancer recurrence [4]. BCS cases with positive margin status as determined by histopathology necessitate follow-up surgery. Standard of care BCS procedures result in positive margins in up to 35% of patients [5], leading to re-excision, where higher positive margin rates are seen at centers where larger normal tissue borders around tumor are required. Additionally, in many BCS patients with adequate tumor margins, negative margins are achieved at the expense of excessive resection of healthy breast tissue with lumpectomy volumes up to 2.5x greater than the necessary resection volume [6]. Not only does follow up surgery delay adjuvant therapy, but it also leads to increased patient stress giving way to an increase in patient morbidity and mortality rates [7–11]. The implementation of intraoperative margin detection methodologies based on pathology techniques including touch prep cytology [12] and frozen section analysis (FSA) [13] have been successful in reducing re-excision rates. However, these aforementioned procedures not only increase surgical time and are dependent upon the presence of a pathologist, but they have also been found to contain significant variation in their sensitivity and specificity between studies [14, 15]. Consequently, the majority of US hospitals have not adopted these pathology based techniques for intraoperative margin assessment during BCS, thus warranting a modality that is able to decrease re-excision rates in a rapid platform within the operating room (OR) [16].

Fluorescence guided surgery (FGS) is an optical imaging modality that has become an attractive tool for intraoperative margin assessment due to its real time imaging capabilities. Fluorescent contrast agents for intraoperative cancer margin assessment are under clinical trial for colorectal, pancreatic, bladder, brain, prostate, head and neck, sarcoma, breast, skin, lung, cervical, ovarian, esophageal and renal cancers [17–19]. However, the path to clinical translation is long and expensive as all ongoing clinical trials apply the novel fluorescent contrast agent to the patient, necessitating extensive safety testing. In the case of BCS and other cancer resections, the diseased tissue is removed from the patient and thus could be stained outside of the body, not requiring patient contact. Technologies that are solely executed on the excised patient samples have a much shorter path to Food and Drug Administration (FDA) approval, since safety testing is not required. While other detection methods have been evaluated for resected specimen staining [20–24], this work is focused on the utility of fluorescence. Given the gaining popularity of FGS and the increase use of “back-table” or ex vivo fluorescence evaluation of resected samples that contain fluorescent contrast administered in vivo, “back-table” FGS systems are currently under commercial development [25, 26]. While all fluorescent contrast will be applied to the resected specimens for our proposed studies, these “back-table” systems are an attractive path to clinical translation for application of our fluorescence based dual-probe difference specimen imaging (DDSI) method for ex vivo staining and biomarker quantification.

In previous work, a robust staining protocol was developed that utilized a dual-probe staining cocktail containing a biomarker targeted probe and matched untargeted probe, which were labeled with spectrally-distinct fluorophores to accurately differentiate between benign and malignant tissues. This novel DDSI methodology proved to be both molecularly specific (89%) and extremely sensitive (97%) for differentiation between benign and malignant murine tissues [27]. Furthermore, DDSI has been validated in several cell lines with varying expression levels of epidermal growth factor receptor (EGFR) and human epidermal growth factor receptor 2 (HER2), confirming the receptor specific diagnostic capabilities of the DDSI methodology [27, 28]. However, the validated DDSI protocol required a total of 16 min for tissue processing time to provide diagnostic information. While this time frame is on par with touch prep cytology [29], it is still rather lengthy and untenable if iterative resection and DDSI is required to accurately quantify margin status. The longest steps in our previously published DDSI protocol were the pre-staining blocking step and the post-staining washing step. Thus, herein we examined the diagnostic accuracy of the DDSI method upon shortening both these steps. Receiver operator characteristic (ROC) curve analysis and calculated area under the curve (AUC) values were used as the metric of diagnostic performance and as a comparison to the previously optimized protocol. Ultimately optimization of a clinically relevant DDSI method would enable rapid margin assessment, while preserving tissue integrity for gold standard histopathology, paving the path for ready clinical translation.

## Methods

### Fluorophores & antibodies

The N-Hydroxysuccinimide (NHS) ester form of Alexa Fluor 647 (AF647, Thermo Fisher Scientific, Waltham, MA) and Cy3B (GE Healthcare Life Sciences, Little Chalfont, UK) were purchased and solubilized in anhydrous dimethyl sulfoxide (DMSO) at 10 mM for antibody conjugation. For the targeted probe, Cetuximab (Erbitux, Eli Lilly and Company, Branchburg, NJ, molecular weight [MW] = 152 kDa) was used and for the untargeted probe, Donkey anti-Rabbit IgG (DkRb, Jackson ImmunoResearch, West Grove, PA, MW = 150 kDa) was used.

### Cell lines

The human epidermoid carcinoma cell line A431, human pancreatic adenocarcinoma cell line AsPC-1, and rat gliosarcoma cell line 9L were cultured in DMEM 1x (Gibco/Thermo Fisher Scientific) with 10% fetal bovine serum (FBS, Seradigm, Sanborn NY) and 1% Penicillin-Streptomycin-Glutamine (Thermo Fisher Scientific). All cell lines were a generous gift from Dr. Kimberley Samkoe (Dartmouth College, Hanover, NH). All cell lines were grown to ~ 90% confluence prior to use for xenograft implantation.

### Antibody-Fluorophore conjugation

As previously described [27], the targeted Cetuximab probe was conjugated to AF647, and the untargeted Dk-anti-Rb IgG probe was conjugated to Cy3B. The antibody conjugation reactions to their respective fluorophores are explained briefly as follows. The antibodies were buffer exchanged into 1x PBS at pH 8.0. Then, 1.5 μl of 10 mM AF647 in anhydrous DMSO was added to 220 μL of Cetuximab and 1 μL of 10 mM Cy3B, in anhydrous DMSO, was added to 220 μL of DkRb. The mixtures were vortex for 2 h at room temperature, protected from light. Prior to purification, the antibody-fluorophore mixtures were concentrated in 10 kDa molecular weight cut off (MWCO) spin filters, then purified through a 6 kDa MWCO desalting column using fast protein liquid chromatography (FPLC, Bio-Rad Laboratories, Hercules, CA). Absorbance spectroscopy (SpectraMax M5, Molecular Devices, San Jose, CA) was used to quantify the antibody to fluorophore conjugation ratio by measuring the antibody absorbance at 280 nm (Cetuximab and DkRb extinction coefficient (ε) = 210,000 M^− 1^ cm^− 1^), Cy3B absorbance at 560 nm (Cy3B ε = 130,000 M^− 1^ cm^− 1^), and AF647 absorbance at 650 nm (AF647 ε = 270,000 M^− 1^ cm^− 1^). DDSI staining solution was composed of a mixture of Cetuximab-AF647 and DkRb-Cy3B containing 1x PBS pH 7.4, 0.1% Tween 20 and 1% Bovine Serum Albumin (BSA) at a final concentration 200 nM of each antibody measured by protein concentration.

### Flow Cytometry

Cells were trypsinized, counted, and fixed in 4% paraformaldehyde (PFA) for 10 min. A 3 min permeabilized step (0.5% Triton-X) was followed by 2 × 5 min washes in phosphate buffered saline (PBS) and then 2 × 10^6^ cells/cell line were blocked for 15 min with 5% FBS. Without removing the blocking buffer, the cells were incubated with 5 μg/ml Cetuximab directly conjugated to AF647 (1:1.7 antibody to fluorophore conjugation ratio). Cells were washed 1 × 5 min with PBS + 0.1% Tween 20, followed by 2 × 5 min PBS washes, and finally resuspended in fresh PBS prior to analysis on a Becton Dickinson LSR Fortessa (Becton, Dickinson and Company, Franklin Lakes, NJ). The flow cytometer was configured with a 640–1 (670/30) Cy5 channel to detect AF647. A minimum of 1 × 10^5^ cells were counted for each sample. To quantify EGFR receptor number, Quantum™ Alexa Fluor® 647 molecules of equivalent soluble fluorophore (MESF) beads (Bangs Laboratories, Inc., Fishers, IN) were quantified prior to the cellular samples.

### Mice, Tumor Implantation & Growth

Female athymic nude mice (32–38 day old, Homozygous 490, Charles River Laboratories, Wilmington, MA) weighing 19–21 g were used to grow xenografts of each cell line. All animal studies were approved by the Institutional Animal Care and Use Committee (IACUC) at Oregon Health and Science University (OHSU). All studies were performed in accordance with these approved guidelines and regulations, which fulfill the ARRIVE guidelines for pre-clinical animal studies. Prior to implantation, mice were anesthetized using 200 μL of a 100 mg/kg Ketamine (Hospira Inc., Lake Forest IL) and 10 mg/kg Xylazine (AnaSed, Shenandoah, IA) solution administered by intraperitoneal (IP) injection. The toe pinch method was used to assess depth of anesthesia.

For A431 and AsPC-1 tumor implantation, the lower peritoneal area was prepared, in a sterile field using povidine-iodine (Purdue Products, Stamford, CT). Along the lateral side of each inferior nipple a small incision (3–5 mm) was made, followed by mammary adipose extraction though the incision using forceps. 200 μL of cell suspension (1 × 10^6^ cells) was injected into the mammary adipose. The mammary adipose was then inserted back into the incision site and the incision was sealed with Vetbond™ (3 M, St. Paul, MN). For 9L xenografts, a 200 μL injection of a 1:1 cell suspension (1 × 10^6^ cells) to Matrigel (Corning, Corning, NY) was subcutaneously injected into both hind flanks. The mice were monitored daily for 5–7 days following the procedure to ensure healing of the surgical site and then weekly for tumor growth and overall health.

Tumors were grown until they reached a maximum size of 1.5 cm^3^, which occurred in 2–3 weeks for both A431 and 9L xenografts, and 4–5 weeks for AsPC-1 xenografts. Tumors were resected and bisected resulting in *n* = 6 tumor samples per blocking condition. For every tumor sample, a corresponding adipose sample was resected as the representative benign control tissue. Six blocking conditions were tested in both the A431 and AsPC-1 tumors types and three blocking conditions were tested in the 9L tumor type, where in total *n* = 18 A431, n = 18 AsPC-1 and *n* = 9 9L tumors were used.

### Tumor resection & DDSI staining

Carbon dioxide (CO_2_) asphyxiation followed by cervical dislocation was used to euthanize all mice prior to tumor excision, a method consistent with the recommendations by the panel on euthanasia of the American Veterinary Medical Association. Each replicate of tumor and adipose tissue was blocked, stained, and washed together in a single Eppendorf tube. Six blocking conditions were tested for each xenograft type: no blocking, 2% BSA for 10 min, 5% BSA for 1 min, 5% BSA for 2 min, 10% BSA for 1 min and 10% BSA for 2 min. All BSA solutions were in 1x PBS at pH 7.4. The previously optimized DDSI staining protocol was used [27], briefly explained as follows: 1 mL of 200 nM Cetuximab-AF647 + 200 nM DkRb-Cy3B in 1x PBS, pH 7.4 containing 1% BSA and 0.1% Tween20 was incubated with the tumor and adipose tissues simultaneously for 1 min. The tissue samples were imaged immediately after staining on a glass slide with the tumor cut face oriented towards the light source and camera. Images were collected after 1 min washes in 50 mL of PBS containing 0.1% Tween-20 for up to 5 min.

### DDSI macroscopic imaging

Color and fluorescence images of the tumor and adipose tissues were collected using a previously described custom-built wide field fluorescence imaging system [30], detailed briefly as follows. A QImaging EXi Blue monochrome camera (Surrey, British Columbia, CA) with a removable Bayer filter and a PhotoFluor II light source (89 North, Burlington, VT) made up the macroscopic fluorescence imaging system. For excitation of Cy3B and AF647, the broad band light source was filtered using a 545 ± 12.5 nm or 620 ± 30 nm bandpass excitation filter, respectively. For fluorescence detection of Cy3B or AF647, a 605 ± 35 nm or a 700 ± 37.5 nm bandpass emission filter was placed in front of the camera, respectively. All filters were obtained from Chroma Technology (Bellows Falls, VT). An aliquot of the staining solution was placed in a covered optical well plate (Greiner Bio-One, Monroe, NC) and imaged with each set of tissues for image calibration.

### DDSI image processing

Custom written MatLab code (MathWorks, Natick, MA) was used to generate DDSI maps from the collected targeted and untargeted fluorescence images, described briefly as follows. Average median background signal was calculated from selected regions of interest (ROI) not containing any tissue and subtracted from the entire image. To normalize intensity between fluorescence channels, a region of interest was also selected from the DDSI staining solution calibration sample images in which an average intensity value was calculated. Each pixel value in each fluorescence image was divided by the average intensity value of the ROI from the respective imaging channel’s DDSI staining solution image. Manual masks were created by the user to identify tissue sample ROIs containing tumor or normal tissues based on the white light images, which were then applied to each fluorescence image. DDSI images were calculated as

$$ {I}_{\mathrm{DDSI}}=\left({I}_{\mathrm{Targeted}}-{I}_{\mathrm{Untargeted}}\right)/{I}_{\mathrm{Untargeted}} $$

where *I =* mean signal intensity.

### IHC Staining & Microscopy

Upon completion of the DDSI protocol, each tumor and adipose tissue replicate was flash frozen in optimal cutting temperature (OCT, Fisher HealthCare, Houston, TX) compound to maintain the tissue orientation from the macroscopic tissue imaging studies. All OCT blocks were sent to OHSU’s Histology Shared Resource where blocks were thawed and re-embedded in paraffin, maintaining tissue orientation. Hematoxylin and eosin (H&E) as well as immunohistochemistry (IHC) staining was performed on serial 4 μm section from each tissue block. IHC staining was completed with an EGFR antibody (1:300, EP38Y, ab52894, AbCam, Cambridge, MA) targeted to a different epitope of EGFR to prevent steric hindrance with pre-existing labeling from Cetuximab. A Zeiss AxioScan.Z1 (Carl Zeiss Microscopy GmbH, Jena, Germany) was used to collect bright field images of all IHC and H&E-stained slides at 10x magnification. The automatic tissue recognition feature in the ZEN software (Zeiss) was used to detect ROIs of IHC and H&E slides. Six field of views were used to set the focus map prior to image scanning and tiling.

### Statistical analysis

As previously described, statistical analysis was performed using MatLab [27]. In brief, receiver operator characteristic (ROC) curves, using a two-by-two decision matrix defining true positive, true negative, false positive, and false negative, were applied to the calculated DDSI maps to assess tumor-to-normal tissue diagnostic performance. ROC curves and corresponding area under the curve (AUC) measurements were calculated on a pixel-by-pixel basis with individual pixel values for each tissue type used as the response variable input. A threshold variable was generated with a linearly increasing value from the minimum to maximum pixel intensity value with a number of values 100 times less than the total number of pixel values. The threshold variable was then used to generate the true positive rate (TPR, percentage of tumor pixels greater than the threshold) and false positive rate (FPR, percentage of normal pixels greater than the threshold), ROC curves and corresponding AUC values were generated by plotting these values at each threshold value. Significant differences between blocking conditions and washing times were evaluated using a one-way analysis of variance (ANOVA) with Tukeys Multiple Comparison test. The α value was set to 0.05 for all ANOVA. All ANOVA was performed using GraphPad Prism (La Jolla, CA).

## Results

### Shortening DDSI blocking conditions

**Fig. 1:**
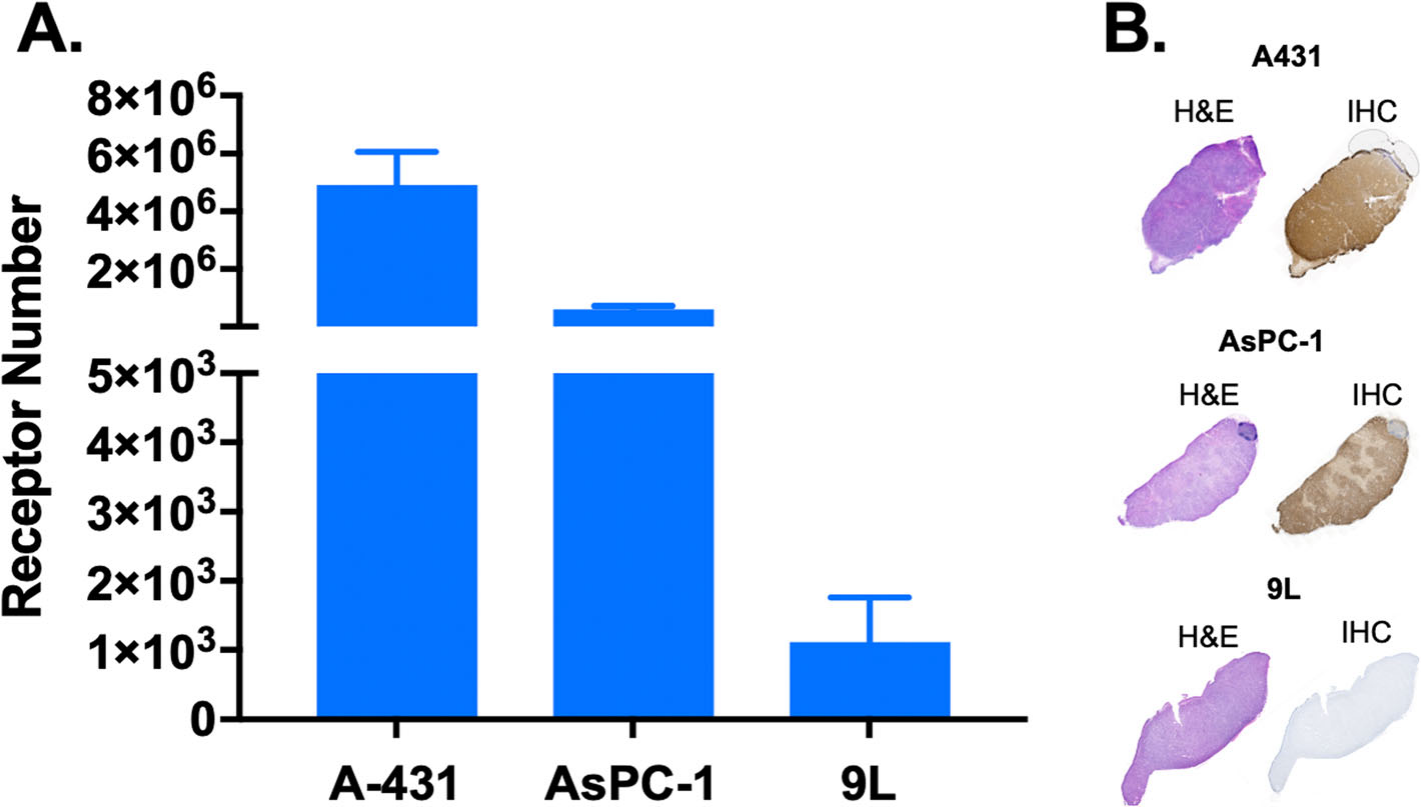
Flow Cytometry Quantification of EGFR status in vitro & IHC validation of EGFR receptor expression ex vivo in the A431, AsPC-1 & 9L cell lines*.* **a** Flow cytometry-based analysis for the A431, AsPC-1 and 9L cell lines was completed for *n* = 3 samples per cell line to quantify EGFR receptor expression. **b** Serial sections of representative resected specimens from each xenograft type (A431, AsPC-1 and 9L) were stained with gold standard H&E and IHC to validate EGFR expression in tissue

**Fig. 2:**
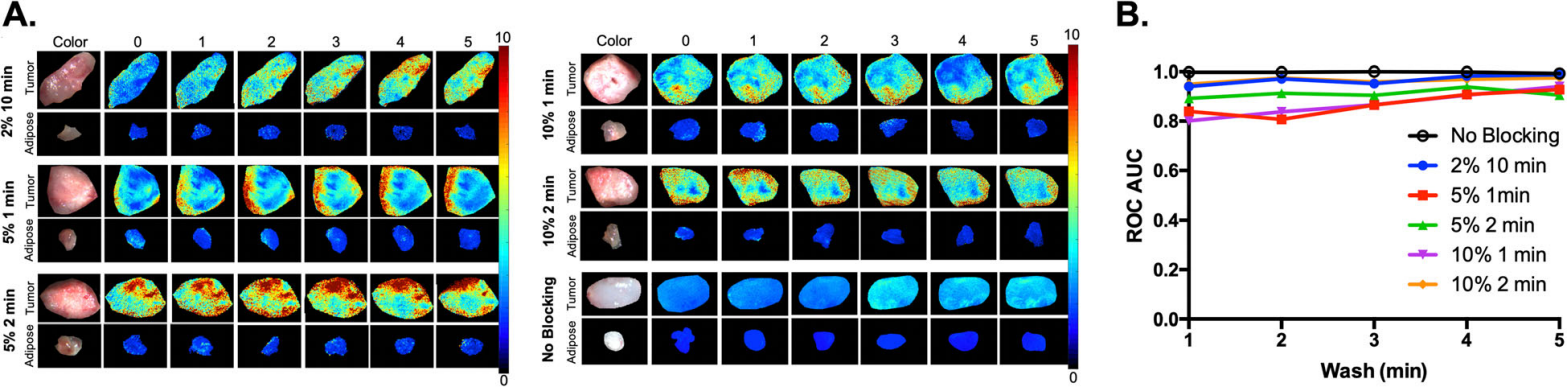
DDSI performance under varying blocking conditions in high EGFR-expressing A431 xenografts*.* **a** Groups of A431 tumors were subject to varied blocking conditions (2% BSA for 10 min, 5% BSA for 1 min, 5% BSA for 2 min, 10% BSA for 1 min, 10% BSA for 2 min and no blocking) to identify the shortest blocking and corresponding washing times that maintained a robust benign and malignant tissue differentiation. All DDSI images are optimally scaled at right (0–10 A.U.) and are representative of *n* = 6 tumor and adipose tissue replicates. **b** The DDSI tumor-to-adipose (T/A) receiver operator characteristic (ROC) curve area under the curve (AUC) values calculated after the varied blocking conditions over washing times were plotted to compare the quantified results

**Fig. 3:**
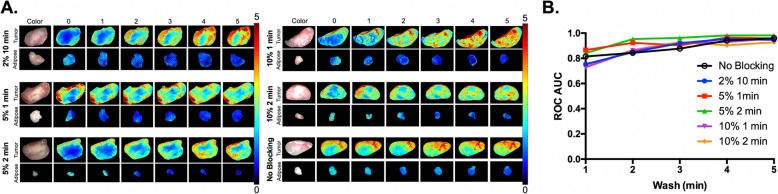
DDSI performance under varying blocking conditions in low EGFR-expressing AsPC-1 xenografts*.* **a** Groups of AsPC-1 tumors were subject to varied blocking conditions (2% BSA for 10 min, 5% BSA for 1 min, 5% BSA for 2 min, 10% BSA for 1 min, 10% BSA for 2 min and no blocking) to identify the shortest blocking and corresponding washing times that maintained a robust benign and malignant tissue differentiation. All DDSI images are optimally scaled at right (0–5 A.U.) and are representative of *n* = 6 tumor and adipose tissue replicates. **b** The DDSI tumor-to-adipose (T/A) ROC AUC values calculated after the varied blocking conditions over washing times were plotted to compare the quantified results

Blocking prior to staining with the targeted and untargeted antibody cocktail has been utilized in our DDSI protocol to decrease non-specific binding in a similar fashion to conventional immunofluorescence staining on tissue sections or cells. Herein, varied percentages of BSA and blocking times were compared to our previously utilized 2% BSA blocking for 10 min to determine if the overall DDSI protocol could be shortened. To permit equivalent non-specific protein accumulation on the tissue, 5% or 10% BSA blocking conditions were tested with shorter incubation times where 1 or 2 min were utilized (*n* = 6 tumor samples per condition). Cell lines with varied EGFR expression in vitro and grown as xenografts were used as model systems for this work, where the high EGFR expressing A431 cell line was compared to the mid-level expressing AsPC-1 cell line and the minimally EGFR expressing 9L cell line (Fig. 1). Minimal difference in DDSI signal intensity or ROC AUC were observed in the highly EGFR expressing A431 tumors (Fig. 2) or the mid-level EGFR expressing AsPC-1 tumors (Fig. 3) when our previous 5 min washing condition was used with any of the tested blocking conditions. To further examine the need for a pre-stain blocking step, DDSI without any BSA blocking was also tested in both the A431 and AsPC-1 tumors. While DDSI without any blocking in the highly EGFR overexpressing A431 tumors showed robust DDSI ROC AUC, the lower EGFR expressing AsPC-1 tumors without blocking showed similar results to all other tested blocking conditions (Figs. 2b and 3b). Comparison of ROC AUC for the tested blocking conditions for A431 and AsPC-1 tumors showed no statistical difference (all *p* values > 0.05).

**Fig. 4:**
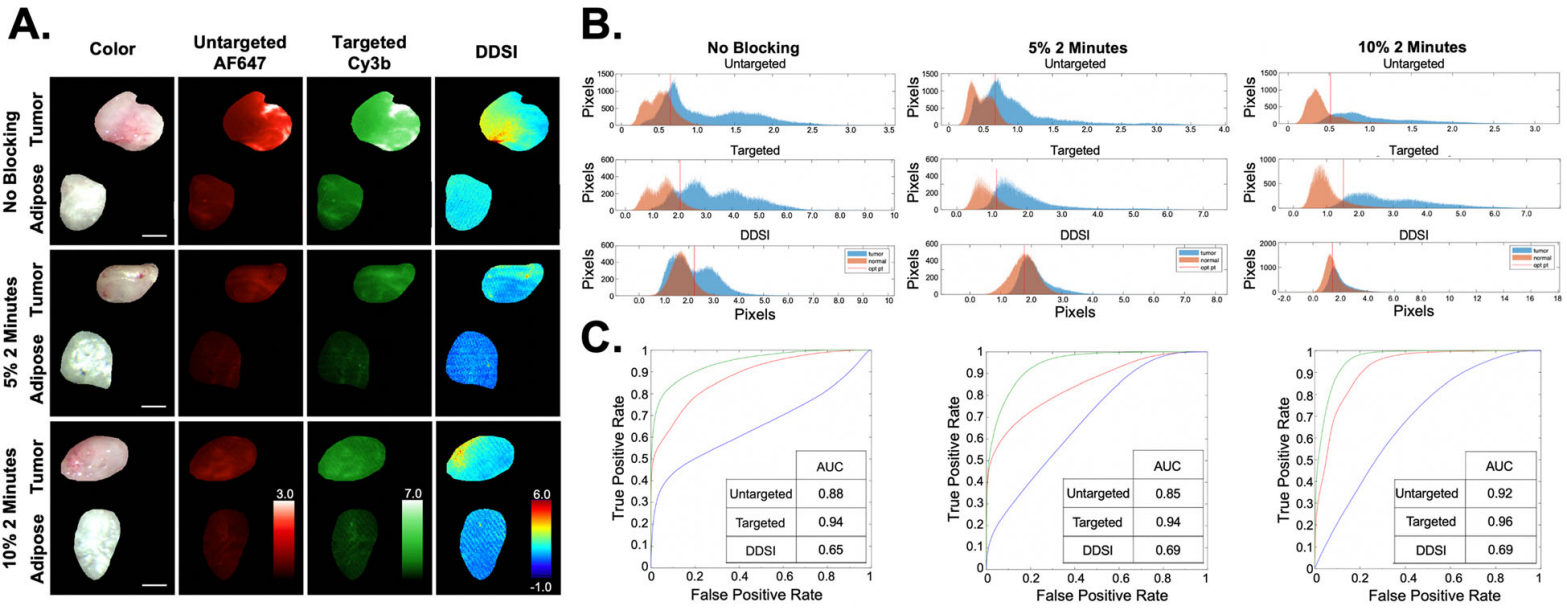
DDSI under varying blocking conditions in EGFR negative 9L xenografts*.* **a** Groups of 9L rat glioma tumors were subjected to the three blocking conditions (no blocking, 5% BSA for 2 min and 10% BSA for 2 min) to validate the robustness of optimized DDSI protocol. **b** Tumor and adipose tissue pixel intensity histograms for the untargeted (top), targeted (middle) and DDSI (bottom) were used to generate **c** ROC curves. AUC values were calculated for that untargeted (red), targeted (green) and DDSI (blue) ROC curves for quantitative comparison between blocking conditions

The DDSI protocol was also evaluated in the EGFR negative 9L cell line using the 10% BSA and 5% BSA for 2 min blocking conditions compared to DDSI without a blocking step. Notably, all tumor and adipose tissue showed uptake of the targeted and untargeted antibody stains (Fig. 4a). However, this non-specific uptake was largely corrected using the ratiometric DDSI protocol. Histograms of pixel intensities showed greater uptake in the benign adipose tissue as compared to the 9L tumor tissue (Fig. 4b). ROC AUC was decreased for DDSI as compared to the targeted and untargeted stains, which was the expected result given the low EGFR expression of the 9L xenografts (Fig. 4c). However, little difference in ROC AUC for DDSI was seen between the three tested blocking conditions. Interestingly, the DDSI pixel intensity histograms showed more complete overlap when using the 5% or 10% BSA blocking conditions as compared to not blocking (Fig. 4b), suggesting that a short blocking step would provide more robust quantification of biomarker expression levels.

### Shortening DDSI washing conditions

Standard DDSI washing conditions were previously set to a 5 min incubation in PBS containing Tween 20 followed by imaging. In an effort to further shorten the staining protocol, DDSI contrast following each minute of washing was evaluated in the highly EGFR expressing A431 and mid-level EGFR expressing AsPC-1 tumors (*n* = 6 tumor samples per condition). Comparison of ROC AUC for the tested washing conditions for A431 and AsPC-1 tumors showed no statistical difference (all *p* values > 0.05). However, DDSI contrast generally improved throughout the 5 min washing period, regardless of the blocking protocol used, particularly for the mid-level EGFR expressing AsPC-1 tumors (Figs. 2a and 3a). DDSI image data was quantified using ROC curve analysis, which showed a similar trend in DDSI ROC AUC values with a generally increased AUC over the 5 min washing period for all tested blocking conditions (Figs. 2b and 3b). Thus, a total washing time of 5 min was maintained in the optimized DDSI protocol for accurate tissue differentiation.

## Discussion

BCS remains the most common treatment for early-stage breast cancer over radical or partial mastectomy [31]. Thus, it is imperative that surgical margins during BCS are negative for cancer in order to decrease patient morbidity and mortality. Current margin detection technologies have several shortcomings that have stifled widespread clinical adoption, including: the requirement of a pathologist within the OR, a staining time not conducive to the clinical workflow, and in some cases the need for patient contact. With the advancement of FGS modalities, the improvement of intraoperative margin assessment has promising potential to reduce positive margins during BCS, which can be as high as ~ 35% [5]. We have developed a fluorescent staining methodology that can be deployed on resected specimens so that neither the contrast agents nor the FGS system require contact with the patient, paving the way for rapid clinical translation.

While our previously published DDSI protocol has high sensitivity and specificity for differentiation between benign and malignant tissues, it requires 16-min of tissue staining time [27], and thus would add substantial OR time if used iteratively to evaluate tumor margins during BCS. Therefore, the overall goal of this study was to develop a tumor margin assessment modality that could be deployed in a relevant time frame for the OR work flow, which was set at < 10 min. The longest components of the previous DDSI protocol were the pre-stain blocking (10 min) and post-stain washing (5 min) steps, where shorter blocking and washing times were investigated herein. This effort was achieved through the use of three tumor cell lines (A431, AsPC-1, and 9L) with varying EGFR expression levels (Fig. 1), alongside benign adipose tissue to ensure retention of similar tumor-to-adipose (T/A) contrast, which is required for successful margin assessment during BCS. Four blocking conditions were selected including 1- or 2-min incubation with a 5% or 10% BSA solution. DDSI contrast using each blocking condition was compared to the previously used 2% BSA blocking for 10 min. Simultaneously, the necessity for a 5 min wash was evaluated by imaging after each minute of washing for all blocking conditions.

The resulting DDSI ROC AUC values for each blocking condition over a cumulative 5-min of washing, showed little variation qualitatively or quantitatively (Figs. 2 and 3). As a result, this brought into question the necessity of a blocking step to maintain a robust DDSI methodology. DDSI without blocking was also tested, and the DDSI ROC AUC was quantified over a 5-min washing period. Surprisingly, the resulting DDSI ROC AUC values for both A431 and AsPC-1 xenografts were similar to the other tested blocking conditions (Figs. 2b and 3b). However, it was notable that the no blocking condition for the highly EGFR expressing A431 tumors resulted in an overall higher uptake of untargeted probe vs. targeted probe, which led to a lower overall calculated DDSI intensity (Fig. 2a).

The necessity of a blocking step was further evaluated by comparing the two most promising blocking protocols (10% or 5% BSA solution incubation for 2 min) vs. not blocking in xenografts from the 9L cell line with minimal EGFR expression. Representative images showed non-specific uptake of both the targeted and untargeted probes in the tumor and benign adipose tissue, but minimal DDSI contrast between the two tissue types as expected (Fig. 4a). The corresponding histograms and ROC curves with calculated AUC values showed that DDSI corrected for non-specific probe uptake with DDSI values ranging from 0.65–0.69, which were a vast improvement over the ROC AUC values for the targeted (0.94–0.96) or untargeted (0.85–0.92) probes alone (Fig. 4c). While the DDSI ROC AUC values showed little difference using the 10% or 5% BSA solution incubated for 2-min vs. not blocking, the histogram pixel intensity values demonstrated improved receptor-specific imaging, as the histograms showed more complete overlap for the normal, benign and tumor tissue using either blocking condition as compared to not blocking (Fig. 4b). This suggested that utilizing a short, high BSA percentage pre-staining blocking step will improve receptor quantification accuracy and result in a more robust DDSI protocol.

Given these results, the 5% BSA for 2-min blocking condition was selected as ideal for the DDSI protocol, based on the overall trends generated from ROC curve analysis (Figs. 2b, 3b, 4b, 4c). Although not blocking demonstrated high AUC T/A values, lower DDSI image intensities signified an increased uptake of the untargeted probe, suggesting that blocking with a low BSA concentration yielded a more robust and ubiquitous DDSI technique. Upon consideration of the washing time necessary for sensitive and specific DDSI ROC curves, the 5-min washing condition was maintained as ROC AUC was generally increased for all blocking conditions for both the A431 and AsPC-1 tumor types for all blocking conditions (Figs. 2 and 3). Thus, the total tissue staining time for this optimized protocol was 8 min (i.e., 2 min blocking, 1 min staining, 5 min washing) decreasing the total tissue staining time by half and fulfilling the goal of a protocol that required < 10 mins. However, if a shorter DDSI protocol was required for clinical work, the washing steps could be shortened without compromising the ability to differentiate tumor from normal tissue using the ROC AUC analysis presented herein as no statistical difference was found as minutes of washing was increased.

The use of DDSI mitigates the need for a pathologist in the operating room and is capable of delivering a rapid diagnosis of the tumor margin intraoperatively without destruction of the resected specimen. Notably, IHC on the pre-surgical breast cancer biopsies could provide a tailored biomarker profile for each patient, enabling personalized DDSI for improved sensitivity and specificity. Preservation of the clinical specimen will ensure post-operative histopathology via gold standard H&E and IHC, which can also be readily co-registered to the corresponding DDSI maps as well as to the margin level pathology of the excised specimens. This will facilitate future validation of the DDSI protocol and comparison to current intraoperative margin pathological methods. In future studies, our optimized DDSI protocol will be translated to human breast cancer specimens, enabling assessment of DDSI contrast against multiple tissues types, where contrast against both adipose and fibroglandular tissues will be imperative for utility to breast cancer margin detection. Since the fundamentals of DDSI rely upon the exploitation of antibody technology, the possibilities to advance this modality to margin detection during surgical intervention for a range of biomarkers and cancer types is a potential future direction for technology development. This could allow for greater diagnostic and surgical intervention in a wide array of cancer types. Limitations of this current study lie in the accuracy of the animal model used since implanted xenografts are homogeneous compared to human cancers. Thus, for full validation, our optimized DDSI protocol warrants translation to ex vivo human specimens for complete validation and testing.

## Conclusions

In summary, the DDSI staining methodology allows for quantitative differentiation between normal, benign and malignant tissues with high diagnostic performance while requiring only 8 min of total tissue processing time. The DDSI methodology possesses several advantages over current tumor margin detection technologies such as touch prep-cytology and FSA, including wide field visualization of the entire tissue specimen with high resolution, and generation of an accurate tumor biomarker expression map, mitigating the need for specialized personnel in the OR and allowing for follow up histopathology in which DDSI generated images can be easily co-registered with conventional H&E and IHC. Ultimately, such an optical imaging modality provides strong potential for rapid intraoperative margin assessment on resected specimens in a clinically relevant time frame, allowing for clinical translation to improve patient outcomes.

## Data Availability

The datasets used and/or analyzed during the current study are available from the corresponding author on reasonable request.
